# MDA‐5 activation by cytoplasmic double‐stranded RNA impairs endothelial function and aggravates atherosclerosis

**DOI:** 10.1111/jcmm.12864

**Published:** 2016-04-29

**Authors:** Tobias Asdonk, Martin Steinmetz, Alexander Krogmann, Christine Ströcker, Catharina Lahrmann, Inga Motz, Kathrin Paul‐Krahe, Anna Flender, Theresa Schmitz, Winfried Barchet, Gunther Hartmann, Georg Nickenig, Sebastian Zimmer

**Affiliations:** ^1^ Department of Medicine/CardiologyUniversity of BonnBonnGermany; ^2^ Institute for Clinical Chemistry and Clinical PharmacologyUniversity of BonnBonnGermany

**Keywords:** atherosclerosis, inflammation, Apolipoprotein E, MDA‐5, polyIC, polyA: reendothelialization, endothelium, endothelial dysfunction

## Abstract

Recent studies have highlighted the relevance of viral nucleic acid immunorecognition by pattern recognition receptors in atherogenesis. Melanoma differentiation associated gene 5 (MDA‐5) belongs to the intracellular retinoic acid inducible gene‐I like receptors and its activation promotes pro‐inflammatory mechanisms. Here, we studied the effect of MDA‐5 stimulation in vascular biology. To gain insights into MDA‐5 dependent effects on endothelial function, cultured human coronary artery endothelial cells (HCAEC) were transfected with the synthetic MDA‐5 agonist polyIC (long double‐stranded RNA). Human coronary endothelial cell expressed MDA‐5 and reacted with receptor up‐regulation upon stimulation. Reactive oxygen species formation, apoptosis and the release of pro‐inflammatory cytokines was enhanced, whereas migration was significantly reduced in response to MDA‐5 stimulation. To test these effects *in vivo*, wild‐type mice were transfected with 32.5 μg polyIC/JetPEI or polyA/JetPEI as control every other day for 7 days. In polyIC‐treated wild‐type mice, endothelium‐dependent vasodilation and re‐endothelialization was significantly impaired, vascular oxidative stress significantly increased and circulating endothelial microparticles and circulating endothelial progenitor cells significantly elevated compared to controls. Importantly, these effects could be abrogated by MDA‐5 deficiency *in vivo*. Finally, chronic MDA‐5 stimulation in Apolipoprotein E/toll‐like receptor 3 (TLR3) double^‐^deficient (ApoE^−/−^/TLR3^−/−^) mice‐enhanced atherosclerotic plaque formation. This study demonstrates that MDA‐5 stimulation leads to endothelial dysfunction, and has the potential to aggravate atherosclerotic plaque burden in murine atherosclerosis. Thus, the spectrum of relevant innate immune receptors in vascular diseases and atherogenesis might not be restricted to TLRs but also encompasses the group of RLRs including MDA‐5.

## Introduction

Atherosclerosis is a chronic inflammatory disease of the vasculature 1, and the leading cause of death worldwide 2. Its main manifestations such as myocardial infarction, ischaemic heart failure, peripheral artery disease and progressive stroke are responsible for significant morbidity and mortality 3. Atherosclerotic plaque formation is a complex process that is orchestrated by vascular cells including endothelial and smooth muscle cells, but also by infiltrating cells of the innate and adaptive immune system. These cells are stimulated by microbial and sterile triggers that act as danger signals inducing innate and acquired inflammatory responses and contribute to atherogenesis 4.

Nucleic acids released from endogenous cells and invading pathogens represent an important danger signal and trigger of inflammation in atherosclerosis. Specific classes and motifs of these nucleic acids bind and activate so called pattern recognition receptors (PRRs) and in turn mediate the production of pro‐inflammatory cytokines and growth factors attributed to atherosclerotic plaque formation 4.

Intracellular *PRRs* comprise cytoplasmatic retinoic acid inducible gene‐I (RIG‐I) like receptors (RLRs), and include RIG‐I and melanoma differentiation associated gene 5 (MDA‐5). They consist of 2N‐terminal caspase recruitment domains (CARD), an DExD/box helicase domain and a C‐terminal repression domain 5. Retinoic acid inducible gene‐I detects RNA with a triphosphate group at the 5′ end 6, 7, whereas MDA‐5 is activated by long double‐stranded RNA in the cytoplasm 8, 11. Activation by exogenous RNA induces a signalling cascade involving interferon‐beta promoter stimulator 1 (IPS‐1, also known as VISA, MAVS and CARDIF) 9, and nuclear transcription factors NF‐κB and IRF‐3, resulting in type I interferon (IFN) production and promotion of an innate immune response. However, details of the underlying cellular interactions and signalling pathways, which may ultimately lead to atherosclerosis, have not been fully deciphered.

Endothelial integrity is indispensable for vascular homeostasis, and the balance of fibrinolysis and coagulation. Furthermore, the endothelial layer directs diapedesis of activated circulating immune cells to sites of inflammation through expression of adhesion molecules, and can be regarded as part of the immune systems itself. In atherosclerosis, endothelial dysfunction is a main feature which persists over all stages 10. The repair of damaged endothelium is promoted by neighbouring endothelial cells (ECs) and – at least indirectly through paracrine activity – by circulating endothelial progenitor cells (EPCs). Endothelial progenitor cells are mobilized from the bone marrow, and their numbers in peripheral circulation correspond with endothelial health 11, 12. Endothelial cells and EPCs express various PRRs, but their exact role in the innate immune mechanisms involved in atherosclerosis still remains poorly understood.

Recently, we demonstrated that activation of RIG‐I in human and murine ECs and EPCs induces pro‐inflammatory effects and may therefore play a crucial role in endothelial biology and provoke atherogenesis 13. Furthermore, double stranded RNA leads endothelial dysfunction *via* endocytosis and toll‐like receptor 3 (TLR3) signalling 14, which is another type of PRR and independent of RLR.

In this study, we sought to examine specific MDA‐5 stimulation of vascular cells on endothelial function, gain novel insights on the development of vascular disease and link for the first time MDA‐5 to atherosclerosis.

## Material and methods

### *In vitro* experiments

Approval for the use of the human buffy coats was granted by the university ethics review board and in accordance with the Declaration of Helsinki.

#### Human coronary artery cells

Human coronary artery endothelial cells (HCAEC; Lonza, Basel, Switzerland) were cultured at 37°C and 5% CO_2_ atmospheric concentration. Endothelial cell growth medium (MU, Promo Cell, Heidelberg, Germany) was renewed every 2 days and cells grown to 80% confluence. For stimulation cells were incubated with polyIC or vehicle polyA with 1 μg/ml. For transfection, Lipofectamine 2000 (Invitrogen, Darmstadt, Germany) was used according to manufacturer's instructions. For TLR3 inhibition, TLR3/Double Strand RNA Complex Inhibitor (Calbiochem/Merck Millipore, Darmstadt, Germany) was added to cell culture as indicated in the Figures S1–S3.

#### Endothelial progenitor cells

Mononuclear cells were isolated from human buffy coats utilizing density Ficoll gradients and circulating angiogenic cells (CAC) were cultured using endothelium cell basal medium‐2 (EBM‐2; Clonetics, Lonza, Basel, Switzerland), as previously described 15. Experiments were performed on day seven after isolation. For stimulation cells were incubated with polyIC or vehicle polyA in concentrations of 1 μg/ml. For transfection Lipofectamine 2000 (Invitrogen) was used according to manufactures instructions. For analysis of early (CFU‐Hill) and late EPCs, mononuclear cells were isolated from murine spleen and seeded into Fibronectin (Sigma Aldrich, Taufkirchen, Germany) pre‐coated 24 well plates at 1 × 10^7^ cells/ml. After 2 days, 1 × 10^6^ non‐adherent cells were cultured for another 7 days. CFU‐Hill were counted using light microscopy. For analysis of late EPCs, the adherent cells were cultured changing EBM‐2 medium every other day. On day 21 cells were regarded late EPCs and quantified using AxioVision version 4.5.0 software (Zeiss, Oberkochen, Germany) and a Zeiss Axiovert 200 M microscope.

#### Immunohistochemistry

Cells were grown in 24‚ well plates on glass slides. For detection of MDA‐5 expression, immunohistochemical stains were performed. Briefly, cells were fixed with acetone at −20°C. They were then washed twice in PBS plus 0.025% Triton X‐100 for 5 min. and subsequently incubated in 1% bovine serum albumin (BSA) with PBS for 2 hrs at room temperature. Next, the primary antibody (Rabbit polyclonal MDA‐5 antibody, ab69983; Abcam, Cambridge, UK) was diluted 1:1000 in 1% BSA with PBS and the slides allowed to incubated overnight at 4°C. Immunodetection was accomplished using an TRITC‐conjugated goat anti‐rabbit secondary antibody (1:1000 dilution). The nuclei were stained with DAPI mounting medium.

#### Real‐time PCR

For analysis of gene expression in cultured HCAEC and EPC, cells were lysed using a 10G needle and homogenized with a motorized homogenizer. RNA was isolated with peqGOLD RNA‐Pure (peqLAB Biotechnology, Erlangen, Germany). RNA concentration and quality was verified with a spectrophotometer. Then, 1 μg of the isolated total RNA was reversely transcribed using Omniscript RT Kit (Qiagen, Hilden, Germany) according to the manufactures protocol. The single‐stranded cDNA was amplified by real‐time quantitative RT‐PCR with the TaqMan System (ABI‐7500 fast PCR System, Thermo Fisher Scientific, Darmstadt, Germany) using SYBR‐Green dye (MDA‐5: forward 5′‐TGG TCT CGT CAC CAA TGA AA‐3′, reverse 5′‐CTC CTG AAC CAC TGT GAG CA‐3′; ICAM‐1: forward 5′‐CGC AAG GTG ACC GTG AAT GT‐3′, reverse 5′‐CGT GGC TTG TGT GTT CGG TT‐3′; VCAM‐1: forward 5′‐AGT CAG GAA TTT CTG GAG GAT GC‐3′, reverse 5′‐GCA GCT TTG TGG ATG GAT TCA C‐3′). For quantification, mRNA expression were normalized to endogenous 18s rRNA.

#### Measurement of reactive oxygen species *in vitro*

For analysis of cellular reactive oxygen species (ROS) production HCAEC and EPC were cultured as described above. Experiments were performed after 2 hrs stimulation. Cells were then trypsinated, pelleted, resuspended in Krebs‐HEPES buffer with 100 μmol/l L‐012 and immediately placed in the scintillation counter.

#### Apoptosis

To determine HCAEC and EPC apoptosis, the Cell Death Detection ELISA Kit (Roche Diagnostics, Mannheim, Germany) was used according to manufacturer's instructions. The results are shown in relative proportion to the control group.

#### Proliferation

Human coronary artery endothelial cells proliferation was assessed by detection of bromodeoxyuridine (BrdU) incorporation after a 24 hrs incubation with polyIC and BrdU. Bromodeoxyuridine positive nuclei were determined using an anti‐BrdU antibody (Abcam) and the total number of cells using DAPI staining.

#### Migration

Endothelial scratch assays were performed as previously described 13. Briefly, ECs were grown to confluence and incubated with polyIC or vehicle polyA with 1 μg/ml cell culture medium. For transfection Lipofectamine 2000 (Invitrogen) was used according to manufactures instructions. The scratch was performed with a sterile pipette and then a marked position was photographed every 2 hrs for 10 hrs by using AxioVision version 4.5.0 software (Zeiss) and a Zeiss Axiovert 200 M microscope. The remaining cell free area was measured and then correlated with the initially scratched area (in percent).

#### Cytokine quantification

Human coronary artery endothelial cells were incubated with polyIC for 24 hrs, and the concentration of IL‐6 and IFN‐γ–induced protein 10 (IP‐10) was then quantified by ELISA in the supernates. Commercially available kits for human IL‐6 and IP‐10 (R&D Systems, Wiesbaden, Germany) were used according to the manufactures protocols. The experimental setup was part of a large screening assay and data from the control group have been published previously 13.

### Animal studies

#### Animals and surgical procedures

For this study, eight to twelve weeks old wild‐type mice (C57BL/6J; Charles River, Sulzfeld, Germany), MDA‐5^−/−^, and ApoE^−/−^TLR‐3^−/−^ mice (C57Bl6 background) were used. The experiments were performed conform to the guidelines from Directive 2010/63/EU, in accordance with institutional guidelines and the German animal protection law. All animals were kept at 22°C in a 12 hrs day/night rhythm and received food and water *ad libitum*. For acute injury experiments wild‐type and MDA‐5 ‐/‐ mice were injected intravenously with either polyIC or polyA, respectively, at doses of 32.5 μg suspended in 200 ml 0.9% NaCl solution every other day for 7 days. Transfection was accomplished with *in vivo* JetPei agent (Polyplus Transfection, Illkirch, France). For chronic injury experiments ApoE^−/−^/TLR‐3^−/−^ mice were fed a high‐fat, cholesterol‐rich diet, that contained 21% fat, 19.5 casein and 1.25% cholesterol for a total of 7 weeks. Mice were injected with polyIC and polyA as described above once a week from week 1 to 5 and then every other day during the last 2 weeks of diet. Aortic segments, hearts, spleen and blood were collected immediately after sacrifice.

For carotid artery injury experiments, wild‐type mice were exposed intravenously to polyIC and polyA every other day during 10 days. The operation was performed on day 5 and animals killed on day 10.

For surgical interventions, mice were intraperitoneally injected ketaminehydrochloride (150 mg/kg body weight; Ketanest; Pharmacia, Erlangen, Germany) and xylazinehydrochloride (10 mg/kg body weight; Rompun 2%; Bayer, Leverkusen, Germany). For sacrifice, mice were anaesthetized with ketamine/xylazine and then killed by cervical dislocation or bleeding.

#### Measurement of reactive oxygen species

Reactive oxygen species release in intact aortic segments was determined by L‐012 chemiluminescence, as previously described 16. Aortic segments were carefully excised and placed in chilled, modified Krebs‐HEPES buffer. Connective tissue was removed and aortas were cut into 2 mm segments. Chemiluminescence of aortic segments was assessed in scintillation vials containing Krebs‐HEPES buffer with 100 μmol/l L‐012 over 15 min. in a scintillation counter (Lumat LB 9501; Berthold, Bad Wildbad, Germany) in 1 min. intervals. The vessel segments were then dried and dry weight was determined. Reactive oxygen species release is calculated as relative chemiluminescence per mg aortic tissue and as percent of control.

#### Flow cytometry

Murine blood samples were analysed as described previously 17. Following red cell lysis, the viable lymphocyte population was analysed for Sca‐1 (Becton Dickinson) and flk‐1 (Becton Dickinson, Heidelberg, Germany) to measure EPC and AnnexinV (Becton Dickinson) and CD 31 (Becton Dickinson) to measure endothelial microparticles (EMP). Isotype identical antibodies and unstained samples served as controls in every experiment (Becton Dickinson). Cell fluorescence was measured immediately after staining using a FACS Calibur instrument (Becton Dickinson). Data were analysed using CellQuest software (Becton Dickinson). Units of all measured components are specific events obtained after measuring 50,000 events (EPC) and 20,000 events (EMP) in a pre‐specified gate during FACS analysis.

#### Aortic ring preparations and tension recording

Vasodilation and vasoconstriction of isolated aortic ring preparations were determined in organ baths filled with oxygenated modified Tyrode buffer (37°C), as previously described 16, 18. Adventitial tissue was carefully removed, and 3 mm segments of the thoracic aorta were investigated. A resting tension of 10 mN was maintained throughout the experiment. Drugs were added in increasing concentrations in order to obtain cumulative concentration–response curves: KCl 20 and 40 mmol/l, phenylephrine 1 nmol/l to 10 μmol/l, carbachol 10 nmol/l to 100 μmol/l (assessment of endothelium‐dependent vasodilation after pre‐contraction with phenylephrine), and nitroglycerin 1 nmol/l to 10 μmol/l (assessment of endothelium‐independent vasodilation after pre‐contraction with phenylephrine). The drug concentration was increased when vasoconstriction or ‐relaxation was completed. Drugs were washed out before the next substance was added.

#### Carotid artery injury

Electrical carotid artery denudation was performed on day 5 of polyIC treatment prior to the third injection. A small incision from the cranial apex of the sternum to just below the mandibule was made. After careful preparation of an approximately 6‐mm‐long segment proximal of the bifurcation, the common carotid artery was electrically denuded applying two serial 5 sec. bursts of 2 Watt using a 2–mm‐wide forceps resulting in a 4 mm lesion. Afterwards the skin was sutured and all mice allowed to recover in individual cages before returning to their littermates. On day 10 of intravenous polyIC treatment, 50 μl Evans Blue were injected intravenously and allowed to circulate for 5 min., mice killed and both common carotid arteries fully excised. The vessels were rinsed in 0.9% NaCl solution and the residual connective tissue carefully removed. Photographs were taken and the remaining denuded area (stained blue) correlated with the total lesion area (4 mm) to measure re‐endothelialization. Images were taken by using AxioVision version 4.5.0 software and Axiovert 200 M microscope (Zeiss).

#### Atherosclerotic plaques, Oil red O and CD68 staining

Hearts with ascending aorta were collected and embedded at −80°C. Samples were sectioned on a Leica cryostat (7 μm) starting at the apex and progressing through the aortic valve area into the ascending aorta and the aortic arch, and placed on slides. Aortic cryosections were then fixed with 3.7% formaldehyde for 1 hr, rinsed with deionized water, stained with oil red = working solution (0.5%) for 30 min. and rinsed again. Haematoxylin staining was performed according to standard protocols. For CD68 staining, rat anti‐mouse CD68 (Acris Antibodies; dilution 1:100) was used as primary antibody, and a Cy3‐conjugated goat anti‐rat secondary antibody (dilution 1:200). Cell nuclei were stained with DAPI. All sections were examined using a Zeiss Axiovert 200 M microscope and AxioVision version 4.5.0 software. To quantify atherosclerotic plaque formation in the aortic root, total area and lipid stained area of serial histological sections were measured. Atherosclerotic data are expressed as lipid‐stained area correlated with total surface area in percent.

### Statistical analysis

Data are presented as mean ± S.E.M. For statistical analysis, two‐tailed, unpaired Student's *t*‐test and anova for multiple comparisons were employed where applicable. *P* < 0.05 indicates statistical significance.

## Results

### Cytoplasmic polyIC increases MDA‐5 expression in HCAECs *in vitro*, and leads to endothelial dysfunction

First we investigated whether HCAECs express MDA‐5. HCAECs constitutively expressed MDA‐5 (Fig. S1A), and specific cytoplasmic stimulation with polyIC – a synthetic double‐stranded RNA analogue on – led to up‐regulation of the receptor (Fig. S1B: polyIC 10.25 ± 2.66/1000 CPB transcripts *versus* polyA 2.25 ± 0.48/1000 CPB transcripts, *P* < 0.05, *n* = 4). Furthermore, MDA‐5 activation was associated with augmented production of ROS (Fig. S1C: polyIC 353.9 ± 115.6% *versus* polyA 100.0 ± 20.50%, *n* = 5, *P* < 0.05) and increased apoptosis (Fig. S1D: polyIC 145.7 ± 8.98% *versus* polyA 100.0 ± 5.85%, *n* = 8–9, *P* < 0.05).

**Figure 1:**
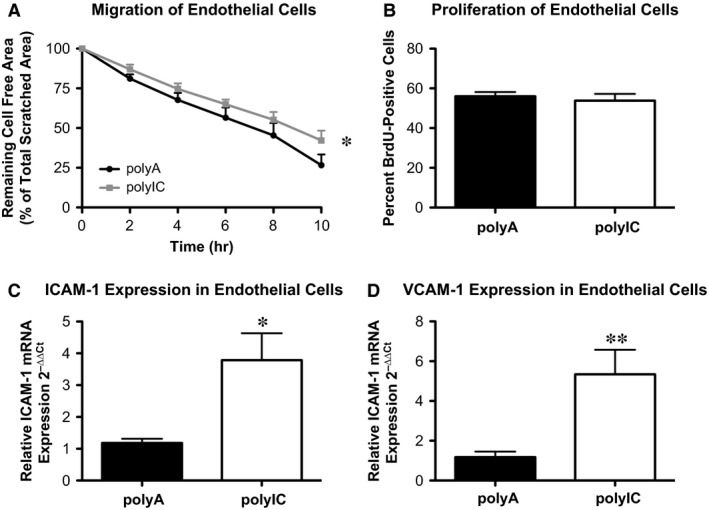
Intracellular delivery of poly IC leads to the expression of markers for endothelial dysfunction. Human coronary artery endothelial cells *in vitro*. (**A**) EC migration after scratch with polyA (control) or poly IC stimulation (*n* = 4–5). (**B**) Proliferation of ECs with polyA (control) or poly IC stimulation (*n* = 4–5). (**C**) Intercellular adhesion molecule 1 (ICAM‐1) and (**D**) vascular cell adhesion molecule 1 (VCAM‐1) expression in ECs with polyA (control) or poly IC stimulation (*n* = 3–5); *P ≤ 0.05, **P ≤ 0.01.

Upon MDA stimulation, endothelial migratory capacity was significantly inhibited (Fig. 1A: polyIC 42.24 ± 2.74% *versus* polyA 26.60 ± 3.40% cell‐free area of total scratched area, *P* < 0.05, *n* = 4–5, and Video S1), whereas proliferation remained unchanged *in vitro* (Fig. 1B: polyIC 53.86 ± 3.35% *versus* polyA 56.06 ± 2.11% of cells positive for BrdU, *P* > 0.05, *n* = 4–5).

Melanoma differentiation associated gene 5 stimulation in HCAEC led to an up‐regulation of ICAM‐1 (Fig. 1C: polyIC 3.79 ± 0.85 2^−∆∆Ct^ *versus* polyA 1.18 ± 0.13 2^−∆∆Ct^, *P* < 0.05, *n* = 3–4) and VCAM‐1 (Fig. 1D: polyIC 5.35 ± 1.23 2^−∆∆Ct^ *versus* polyA 1.18 ± 0.28 2^−∆∆Ct^, *P* > 0.05, *n* = 3–5), two adhesion molecules associated with activated and dysfunctional ECs 19.

**Figure 2:**
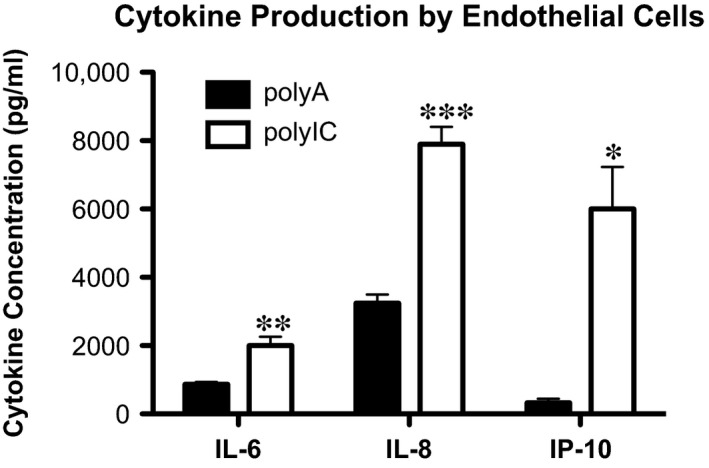
Intracellular delivery of poly IC leads to the production of pro‐inflammatory cytokines IL‐6, IL‐8, and IP‐10. Human coronary artery endothelial cells *in vitro*. Interleukin‐6 (IL‐6), interleukin‐8 (IL‐8) and interferon γ‐induced protein 10 (IP‐10) in the supernatant of ECs with polyA (control) or polyIC stimulation (*n* = 4), detected by ELISA; ***P ≤ 0.001.

Finally, stimulation of MDA‐5 was associated with the release of pro‐inflammatory cytokines IL‐6 (Fig. 2: polyIC 2000 ± 260.3 pg/ml *versus* polyA 866.8 ± 69.71 pg/ml, *P* < 0.05, *n* = 4), IL‐8 (Fig. 2: polyIC 7898 ± 502.6 pg/ml *versus* polyA 3248 ± 251.9 pg/ml, *P* < 0.05, *n* = 4), und IP‐10 (Fig. 2: polyIC 6001 ± 1228 pg/ml *versus* polyA 327.3 ± 117.9 pg/ml, *P* < 0.05, *n* = 4). Taken together, these results not only demonstrate immune competence of ECs in response to cytosolic long double‐stranded RNA, but also represent hallmarks of endothelial dysfunction.

### MDA‐5 activation induces endothelial dysfunction in wild‐type mice

On the basis of our *in vitro* data, we next investigated whether MDA‐5 activation influences endothelial biology in wild‐type mice. PolyIC or control RNA (polyA) were supplemented with JetPEI for cytoplasmic transfection, and C57Bl6J mice injected intravenously every other day for 7 days. Similar to packaging polyIC with Lipofectamine (Invitrogen) for transfection *in vitro*, conjugation of polyIC with jetPEI (Polypus) mediates activation MDA‐5 *in vivo* through its transfection to the cytoplasm.

**Figure 3:**
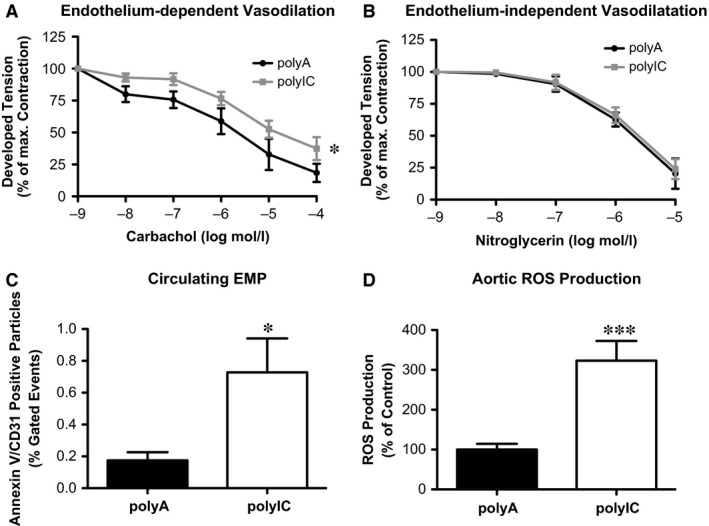
Intracellular delivery of poly IC *in vivo* promotes endothelial dysfunction in C57bl6J mice. (**A**) endothelium‐dependent and (**B**) independent vasodilation with polyA (control) or polyIC stimulation. (**C**) Circulating endothelial microparticles (EMP). (**D**) Reactive oxygen species of thoracic aorta; each *n* = 5, *P ≤ 0.05, ***P ≤ 0.001.

First, regulation of vascular tension by aortic rings, as a measurement of endothelial function, was investigated in organ chamber experiments. Endothelium‐dependent vasodilation was significantly impaired in mice transfected with polyIC, which marks endothelial dysfunction (Fig. 3A: polyIC 37.42 ± 8.96% *versus* polyA 18.47 ± 7.14% of maximal contraction, *n* = 5, *P* < 0.05). On the contrary, phenylephrine‐induced vasoconstriction (data not shown) and endothelium‐independent vasodilation through nitroglycerin (Fig. 3B: polyIC 23.91 ± 7.84% *versus* polyA 20.41 ± 11.88% of maximal contraction, *P* > 0.05, *n* = 5) showed no differences between the two groups, suggesting that vascular smooth muscle cells were fully functional and intact after polyIC treatment.

The number of circulating EMPs which are released by dysfunctional ECs and therefore a biomarker for endothelial dysfunction, was significantly increased compared to control mice (Fig. 3C: polyIC 0.73 ± 0.21% *versus* polyA 0.17 ± 0.05% gated events, *n* = 5, *P* < 0.05). Reactive oxygen species production of isolated aortic rings was measured by L‐012 chemiluminescence. Melanoma differentiation associated gene 5 stimulation increased vascular oxidative stress compared to control mice (Fig. 3D: polyIC 323.3 ± 49.45% *versus* polyA 100.0 ± 14.52%, *n* = 5, *P* < 0.05).

**Figure 4:**
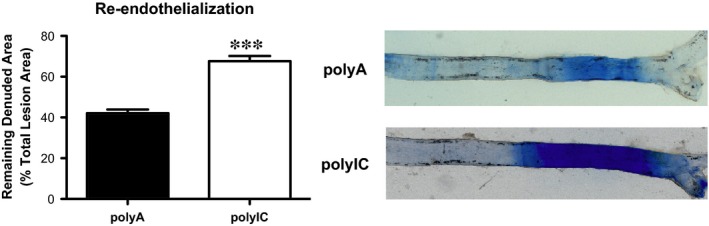
Intracellular delivery of poly IC *in vivo* reduces reendothelialization in C57bl6J mice. Reendothelialization after perielectric injury and staining of defects in the endothelial monolayer with Evan's Blue; *n* = 5‐7, ***P ≤ 0.001.

To assess endothelial regeneration capacity *in vivo,* wild‐type mice were injected intravenously with polyIC or control RNA every other day for a total of 10 days. On day 5 electrical carotid artery injury was performed and a 4 mm segment proximal to the bifurcation was denuded. The rate of reendothelialization was assessed after staining with Evans Blue on day 10. Systemic MDA‐5 stimulation resulted in significantly reduced reendothelialization compared to control (Fig. 4: polyIC 67.62 ± 2.53% *versus* polyA 42.11 ± 1.78% of total lesion area, *P* < 0.05, *n* = 5–7).

### Sca‐1/flk‐1 positive progenitor cell numbers are increased in wild‐type mice with polyIC induced MDA‐5 activation

**Figure 5:**
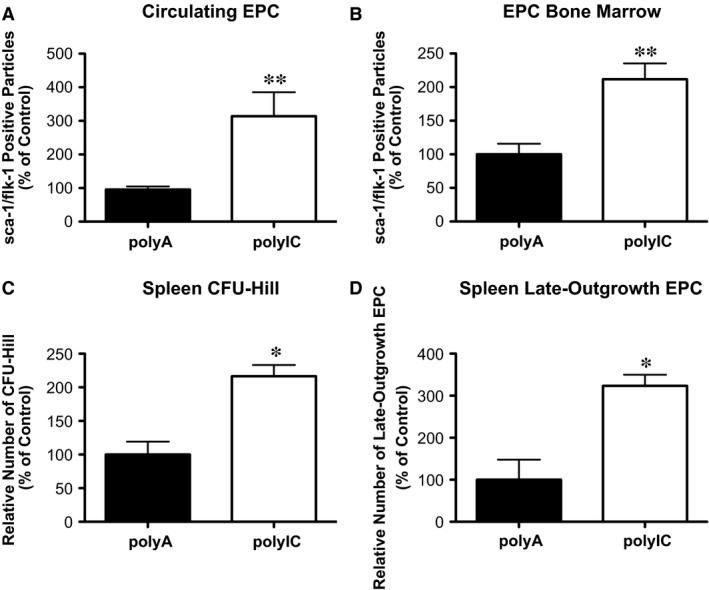
Intracellular delivery of poly IC *in vivo* increases endothelial progenitor cell subsets in C57bl6J mice. Flow cytometric analysis of sca‐1/flk‐1 positive endothelial progenitor cells (EPCs) in (**A**) peripheral blood and (**B**) bone marrow. Histomorphological analysis of vitro EPC subsets: (**C**) colony forming unit Hill (CFU‐Hill) and (**D**) late‐outgrowth EPC;* n* = 5, *P ≤ 0.05, **P ≤ 0.01.

Like EMPs, circulating EPCs are well‐established as a biomarker for to endothelial restoration. Melanoma differentiation associated gene 5 stimulation led to elevated sca‐1/flk‐1 pos. progenitor cells not only in the blood (Fig. 5A: polyIC 314.0 ± 71.19% *versus* polyA 95.43 ± 9.56%, *P* < 0.05, *n* = 5–7) but also in the bone marrow (Fig. 5B: polyIC 211.7 ± 23.59% *versus* polyA 100.0 ± 15.82%, *P* < 0.05, *n* = 5). Spleen‐derived CFU‐Hill (Fig. 5C: polyIC 216.7 ± 16.67% *versus* polyA 100.0 ± 19.25%, *P* < 0.05, *n* = 5) and late‐outgrowth EPCs (Fig. 5D: polyIC 323.6 ± 26.10% *versus* polyA 100.0 ± 47.77%, *P* < 0.05, *n* = 5) were also increased upon systemic MDA‐5 stimulation.

### MDA‐5 knockout abrogates polyIC effects *in vivo*

**Figure 6:**
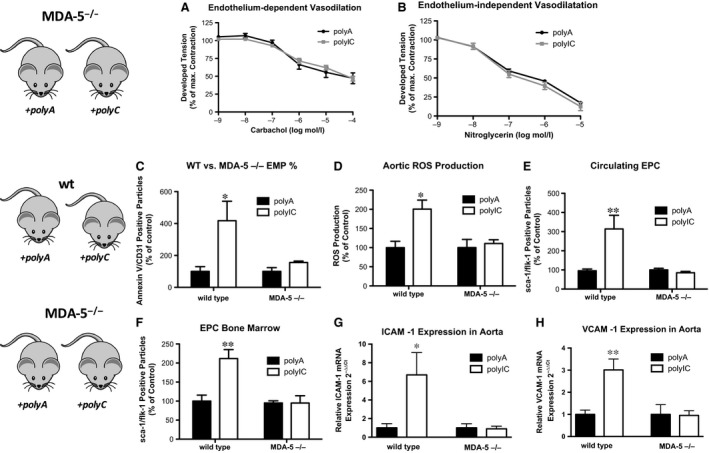
Intracellular delivery of poly IC *in vivo* acts *via* MDA‐5. (**A**) Endothelium‐dependent and (**B**) endothelium‐independent vasodilation in MDA‐5‐/‐ mice with polyA (control) or polyIC. (**C**) Endothelial microparticles, (**D**) production of reactive oxygen species in thoracic aorta in wild‐type *versus* MDA‐5‐/‐ mice with polyA (control) or polyIC. (**E**) Endothelial progenitor cells in peripheral blood (circulating EPC) and (**F**) in the bone marrow of wild‐type *versus* MDA‐5‐/‐ mice with polyA (control) or polyIC. (**G**) ICAM‐1 expression and (**H**) VCAM‐1 expression in the thoracic aorta of wild‐type *versus* MDA‐5‐/‐ mice with polyA (control) or polyIC;* n* = 4–5, *P ≤ 0.05, **P ≤ 0.01.

To verify the dependency of MDA‐5 for the found effects, we treated MDA‐5‐deficient (MDA‐5^−/−^) and wild‐type control mice with either polyA or polyIC. In organ chamber experiments, polyIC treatment of MDA‐5^−/−^ had no effect on endothelium‐dependent vasodilation compared to polyA‐treated MDA‐5^−/−^ mice (Fig. 6A and B: polyIC 46.67 ± 3.34% *versus* polyA 47.20 ± 7.54% of maximal contraction, *P* > 0.05, *n* = 5). Furthermore, EMP numbers (Fig. 6C: polyIC 0.24 ± 0.03% *versus* polyA 0.17 ± 0.04% gated events, *P* > 0.05, *n* = 5) and ROS production (Fig. 6D: polyIC 110.82 ± 9.79% *versus* polyA 100.0 ± 21.41%, *P* > 0.05, *n* = 5) remained unchanged. Similarly, sca‐1/flk‐1 pos. Endothelial progenitor cells in the blood (Fig. 6E: polyIC 85.08 ± 7.33% *versus* polyA 100.0 ± 8.44%, *P* > 0.05, *n* = 5) and in the bone marrow (Fig. 6F: polyIC 95.20 ± 5.62% *versus* polyA 94.83 ± 19.20%, *P* > 0.05, *n* = 5) were not elevated upon systemic polyIC treatment. Finally, ICAM‐1 (Fig. 6H: wild‐type: polyIC 4.99 ± 1.75 *versus* polyA 0.64 ± 0.29, *P* < 0.05; MDA‐5^−/−^: polyIC 0.92 ± 0.31 *versus* polyA 1.03 ± 0.46, *P* > 0.05) and VCAM‐1 (Fig. 6G: wild‐type: polyIC 1.91 ± 0.31 *versus* polyA 0.63 ± 0.12, *P* < 0.05; MDA‐5^−/−^: polyIC 0.87 ± 0.19 *versus* polyA 0.92 ± 0.40, *P* > 0.05) expression were significantly induced upon polyIC treatment in wild‐type control mice, whereas no change of expression was found in MDA‐5^−/−^ mice. Together, these findings underline that endothelial dysfunction is directly mediated by MDA‐5 receptor activation.

### MDA‐5 activation aggravates atherosclerotic plaque burden in ApoE^−/−^ mice

Because endothelial damage is associated with the development and aggravation of atherosclerotic plaques, we investigated the effects of repetitive polyIC induced MDA‐5 activation in murine atherosclerosis. 10–12‐week‐old ApoE/TLR3^−/−^ mice received a cholesterol‐rich, high‐fat diet for a total of 7 weeks. They were concomitantly injected with JetPEI/polyIC or JetPEI/polyA once a week for 5 weeks and thereafter every other day for a further 2 weeks of diet. ApoE/TLR3^−/−^ mice were chosen to exclude TLR3‐effects and extrapolate the role of MDA‐5.

**Figure 7:**
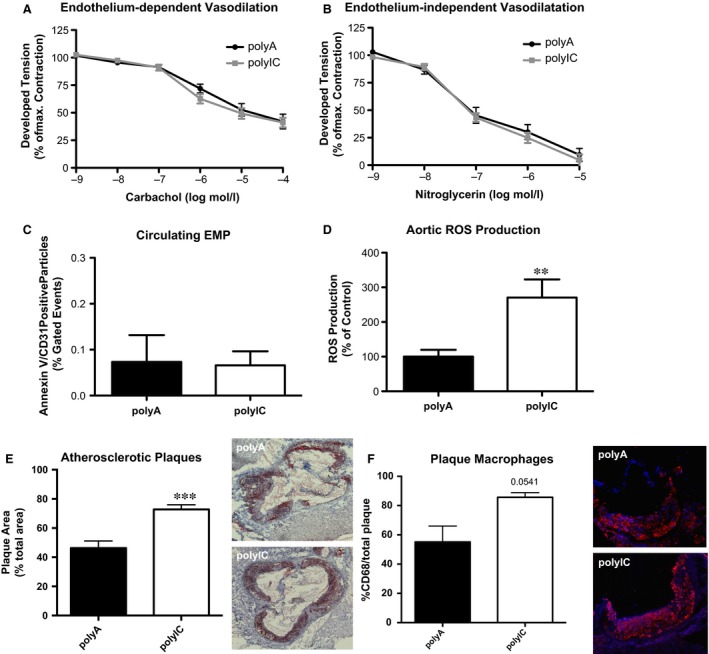
Intracellular delivery of poly IC *in vivo* for 7 weeks increases ROS production and atherosclerotic plaques in ApoE/TLR3‐deficient mice. ApoE/TLR3‐/‐ mice with a cholesterol‐rich high fat diet were injected polyA or polyIC during 7 weeks. (**A**)Endothelium‐dependent and (**B**) independent vasodilation, (**C**) circulating endothelial microparticles (EMP) and (**D**) production of reactive oxygen species (ROS) at sacrifice. (**E**) Oil red O staining for atherosclerotic plaques in the aortic root/ascending aorta (*n* = 7–9). (**F**) Relative content of CD68 positive macrophages in atherosclerotic plaques; **P ≤ 0.01, ***P ≤ 0.001.

To our surprise, chronic MDA‐5 activation did not change endothelium‐dependent (Fig. 7A: polyIC 41.08 ± 4.32% *versus* polyA 42.06 ± 6.64% of maximal contraction, *P* > 0.05, *n* = 7–9) or nitroglycerin‐induced vasodilation (Fig. 7B: polyIC 4.63 ± 5.21% *versus* polyA 9.40 ± 5.82% of maximal contraction, *P* > 0.05, *n* = 7–9). EMPs were not further elevated in polyIC‐treated mice (Fig. 7C: polyIC 0.07 ± 0.03% *versus* polyA 0.07 ± 0.06% gated cells, *P* > 0.05). However, chronic MDA‐5 stimulation was associated with increased ROS formation (Fig. 7D: polyIC 270 ± 52.53% *versus* polyA 100 ± 19.99%, *P* < 0.05, *n* = 7–9).

Finally, the ApoE/TLR3^−/−^ with repetitive polyIC injections showed significantly increased plaque burden in the aortic root compared to polyA controls (Fig. 7E: polyIC 72.79 ± 3.19% *versus* polyA 46.24 ± 4.81% of total area, *P* < 0.05, *n* = 6). Although not significant, there was a trend towards higher macrophage content in mice treated with polyIC (Fig. 7F: polyIC 85.68 ± 3.15% *versus* polyA 55.13 ± 10.87%).

## Discussion

The exact mechanisms for endothelial dysfunction and consecutive vascular inflammation in atherosclerosis are not fully understood. Our results show for the first time that specific stimulation of MDA‐5 induces endothelial dysfunction and can significantly aggravate atherosclerosis, most likely through the enhanced expression of adhesion molecules on EC with subsequent plaque infiltration of macrophages, which is accompanied by oxidative stress. These findings are in line with recent studies that linked RIG‐I 13, and TLR2, 3, 4, 5 and 9 to endothelial deterioration 14, 20, 21, 22 and the orchestration of vascular inflammatory responses. Thus, damaged ECs seem to be not only a by‐product of vascular inflammation, but actively participate in the inflammatory process through activation innate immune sensors such as MDA‐5.

The RLRs MDA‐5 and RIG‐I are mainly known as a virus sensing system that induce the production of type I IFNs by dendritic cells and macrophages. Viral fragments have been found in atherosclerotic lesions 23, 24, and there is a growing body of evidence that recurrent or chronic viral infections worsen atherosclerosis. However, viral infections alone most likely do not cause the development, but rather are a contributing factor for the course of atherosclerosis. A study by Paik and colleagues demonstrated that LDLR^−/−^ mice with norovirus infection had a significantly higher plaque burden 25. Admittedly, the investigators found more macrophage activation in the infected mice, and did not analyse endothelial health or activity. As norovirus is a natural ligand for MDA‐5, this interaction might be similar to our stimulation of ApoE^−/−^ mice with polyIC. According to our study results, endothelial dysfunction could therefore be a complementary mechanism for the detrimental effects of viruses on atherosclerosis through the production of pro‐inflammatory cytokines such as IP‐10, IL6 and IL‐8, and expression of cell adhesion molecules VCAM‐1 and ICAM‐1 upon MDA‐5 stimulation. In humans, viral infections such as influenza are strongly associated with the occurrence of cardiovascular events during the flu season, and matched vaccination proved to have protective effects in patients with coronary artery disease 26.

Nevertheless, recent studies indicate that RLRs are not only involved in antiviral immune response but also contribute to chronic inflammatory disease. Retinoic acid inducible gene‐I activation for instance has been associated with rheumatoid arthritis and lupus nephritis 27, and is highly expressed in intimal macrophages of atherosclerotic lesions 28. Melanoma differentiation associated gene 5 is associated with increased risk of developing type I diabetes 5, 29, and has been described as an auto‐antigen for humoural immune response in myositis 30. However, MDA‐5 had not yet been associated with vascular biology and atherogenesis.

Although specific ligands for most PRR have been identified, numerous PRRs are not restricted to a single exogenous danger signal but rather show cross reactivity to several endogenous molecules such as IFNs, heat shock proteins or oxidized cholesterol 4, 27. One may speculate that nucleic acids from apoptotic or necrotic vascular cells could stimulate RLRs of neighbouring cell and provoke a pro‐inflammatory vascular state that serves as a fertile ground to accelerate vascular inflammation in viral infections.

Retinoic acid inducible gene‐I and MDA‐5 share morphologic homologies and both trigger an innate immune response through a common intracellular signalling cascade. Both stimulate IPS‐1 which in turn activates NF‐κB and IRF‐3 resulting in the production of type I IFN‐β 27. However, some differences in endothelial reaction after specific receptor stimulation can be noted. Melanoma differentiation associated gene 5 activation *in vivo* increased EPCs and *in vitro* induced HCAEC apoptosis, whereas RIG‐I stimulation had no such effects. Slight differences in individual effects seem not surprising given that RIG‐I and MDA‐5 share only ~25% homology within the CARD domain regions and 40% within the helicase domain 5. LGP2 (Laboratory of Genetics and Physiology 2) is a third RLR that has been considered incomplete because of its lack of a CARD domain. Recent data have shown a sensitizing effect of LGP2 on MDA‐5, but not on RIG‐I 31.

The inhibition of PRR mediated vascular inflammation and endothelial dysfunction could benefit future anti‐atherosclerotic therapies. Preventing the multimerization of the RIG‐I CARD domains through the anti‐aging protein Klotho, for example, diminished the release of the pro‐inflammatory cytokines IL‐6 and IL‐8 27.

In summary, our study demonstrates that stimulation of the cytosolic RLR MDA‐5 induces endothelial dysfunction and promotes atherosclerosis. Approaches to inhibit these vascular innate immune processes should be investigated for potential beneficially effects on endothelial dysfunction or even atherogenesis.

## Funding

This study was supported by the Medical Faculty of the Rheinische‐Friedrich‐Wilhelms‐University Bonn (BONFOR) and Deutsche Forschungsgemeinschaft (DFG).

## Disclosure

None.

## Supporting information


**Figure S1** Intracellular delivery of polyIC leads to MDA‐5 induction. (**A**) Representative image (immunofluorescence) of MDA‐5 expression by human coronary artery endothelial cells (HCAEC) *in vitro*. (**B**) Expression of MDA‐5 mRNA in ECAECs upon 24 hrs polyA (r poly C transfection, *n* = 4). (**C**) Production of ROS in HCAEC after polyA or poly C stimulation (*n* = 5). (**D**) Relative apoptosis of ECAEC with polyA (r poly C stimulation, *n* = 8–9); *P ≤ 0.05, ***P ≤ 0.001.
**Figure S2** Intracellular delivery of polyIC in the presence of a TLR3‐antagonist leads to MDA‐5 induction and IP10 release. Human coronary artery endothelial cells *in vitro*. (**A**) Expression of MDA‐5 messenger RNA in ECs with polyA (control) or poly IC stimulation (*n* = 2–3). (**B**) IP10 concentration in the supernatant of stimulated ECs (*n* = 2–3); *P ≤ 0.05, ***P ≤ 0.001; Lipo: Lipofectamine 2000; TLR3 inh.: TLR3/dsRNA complex inhibitor.
**Figure S3** Gating strategies for endothelial microparticles and sca1/flk1 positive progenitor cells. (**A**) EMPs have been identified as CD31 positive and Annexin V positive microparticles. (**B**) To enumerate EPCs, the lymphocyte population was scanned for cells that co‐express sca1 and flk1/VEGFR2. EMP: endothelial microparticle; EPC: endothelial progenitor cell.

**Video S1** Representative video of endothelial cell migration scratch assay. The video is a composition of sequential photographs taken every 3 min. after physically scratching a cell‐free strip. Vehicle (left) and polyIC (right) stimulated cells.
