# Supplementary material for: MDA‐5 activation by cytoplasmic double‐stranded RNA impairs endothelial function and aggravates atherosclerosis
Source: J Cell Mol Med. 2016 Apr 29;20(9):1696–705. doi: 10.1111/jcmm.12864 (PMC4993381; doi:10.1111/jcmm.12864)

Figure S1: Intracellular delivery of poly IC leads to MDA-5 induction

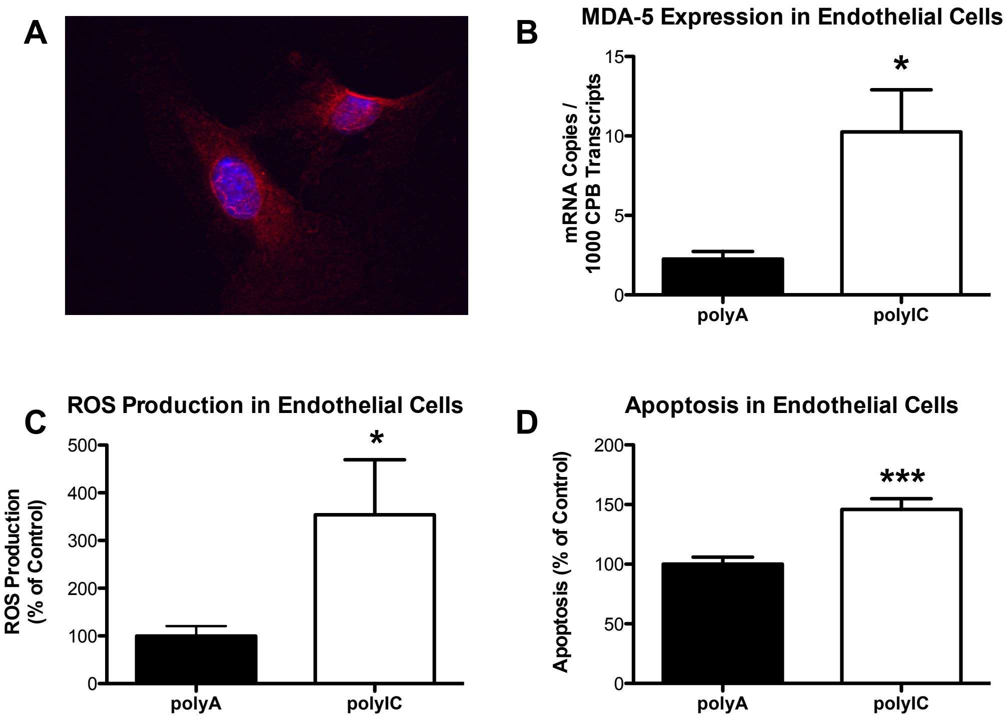

Figure S2: Intracellular delivery of polyIC in the presence of a TLR3-antagonist leads to MDA-5 induction and IP10 release

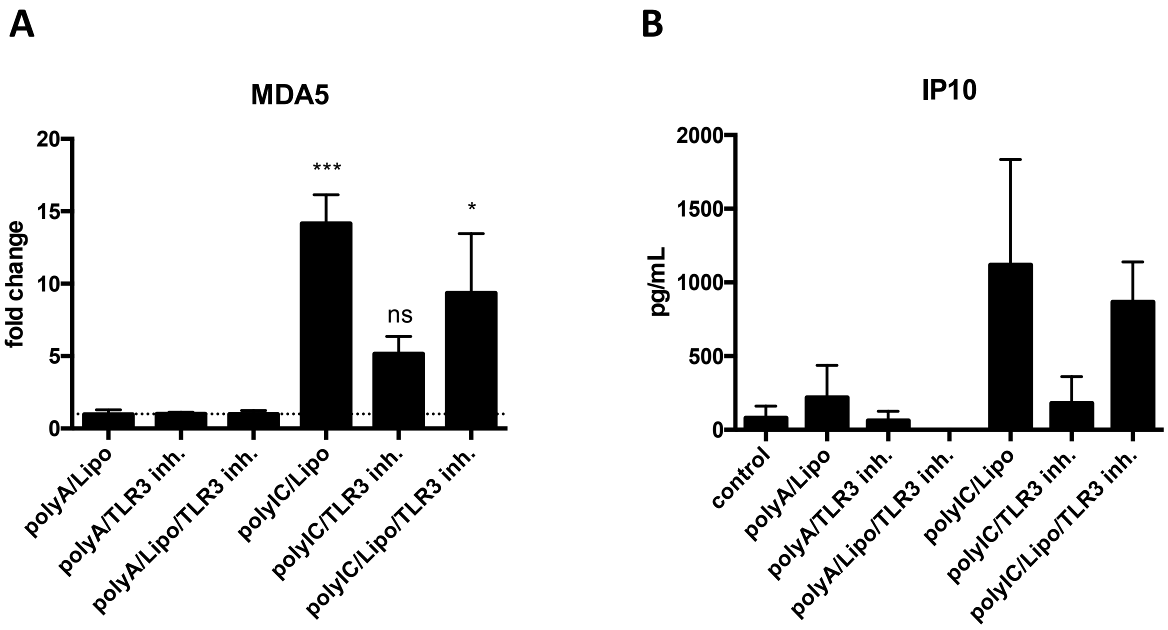

Figure S3: Gating strategies for endothelial microparticles and sca1/flk1 positive progenitor cells

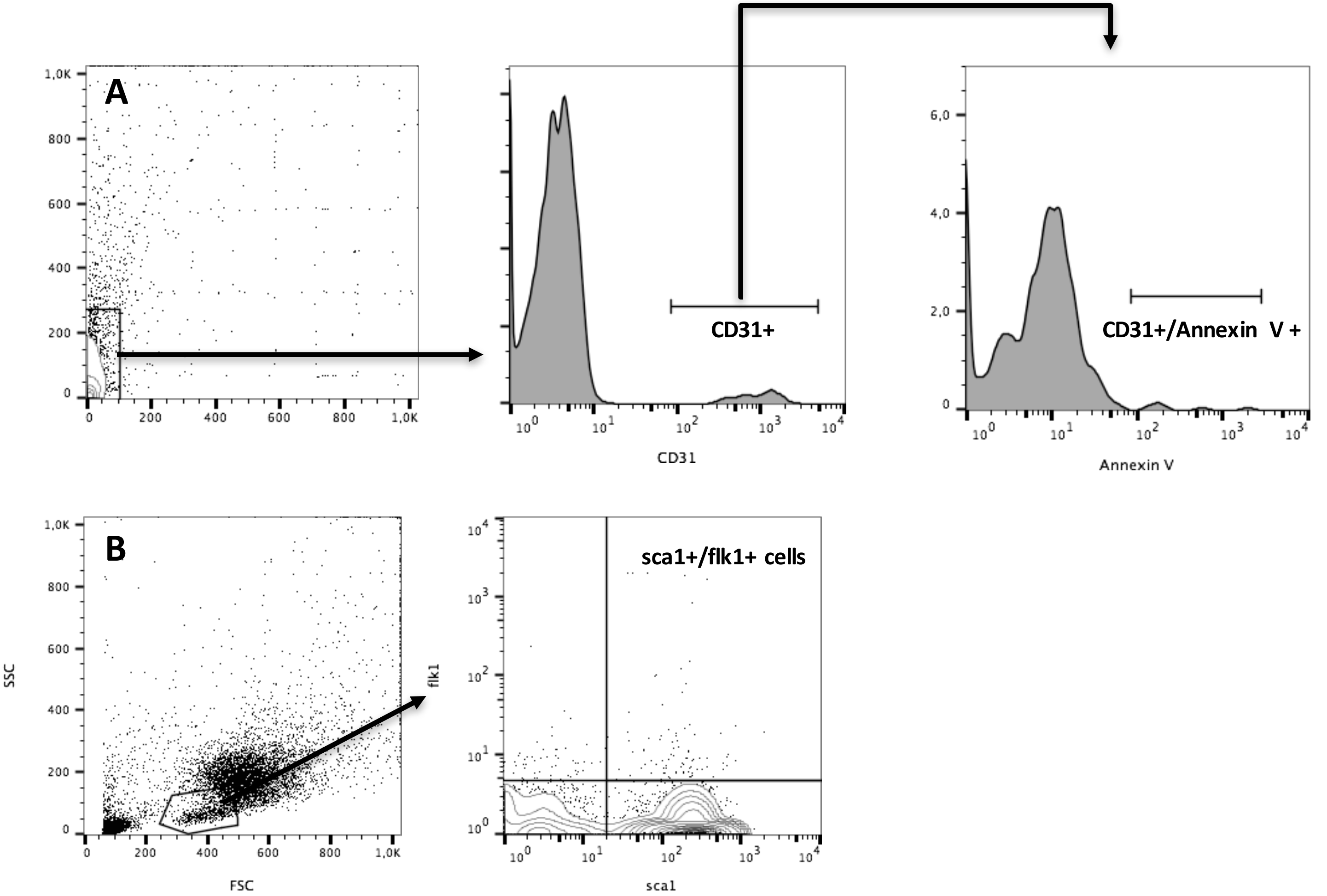

Supplement: Supplementary file 1 — Figure S1 Intracellular delivery of polyIC leads to MDA‐5 induction. (A) Representative image (immunofluorescence) of MDA‐5 expression by human coronary artery endothelial cells (HCAEC) in vitro. (B) Expression of MDA‐5 mRNA in ECAECs upon 24 hrs polyA (r poly C transfection, n = 4). (C) Production of ROS in HCAEC after polyA or poly C stimulation (n = 5). (D) Relative apoptosis of ECAEC with polyA (r poly C stimulation, n = 8–9); *P ≤ 0.05, ***P ≤ 0.001. Figure S2 Intracellular delivery of polyIC in the presence of a TLR3‐antagonist leads to MDA‐5 induction and IP10 release. Human coronary artery endothelial cells in vitro. (A) Expression of MDA‐5 messenger RNA in ECs with polyA (control) or poly IC stimulation (n = 2–3). (B) IP10 concentration in the supernatant of stimulated ECs (n = 2–3); *P ≤ 0.05, ***P ≤ 0.001; Lipo: Lipofectamine 2000; TLR3 inh.: TLR3/dsRNA complex inhibitor. Figure S3 Gating strategies for endothelial microparticles and sca1/flk1 positive progenitor cells. (A) EMPs have been identified as CD31 positive and Annexin V positive microparticles. (B) To enumerate EPCs, the lymphocyte population was scanned for cells that co‐express sca1 and flk1/VEGFR2. EMP: endothelial microparticle; EPC: endothelial progenitor cell. [file JCMM-20-1696-s001.pdf]
