# Supplementary figures and images for: MDA‐5 activation by cytoplasmic double‐stranded RNA impairs endothelial function and aggravates atherosclerosis
Source: J Cell Mol Med. 2016 Apr 29;20(9):1696–705. doi: 10.1111/jcmm.12864 (PMC4993381; doi:10.1111/jcmm.12864)

## Slide 1
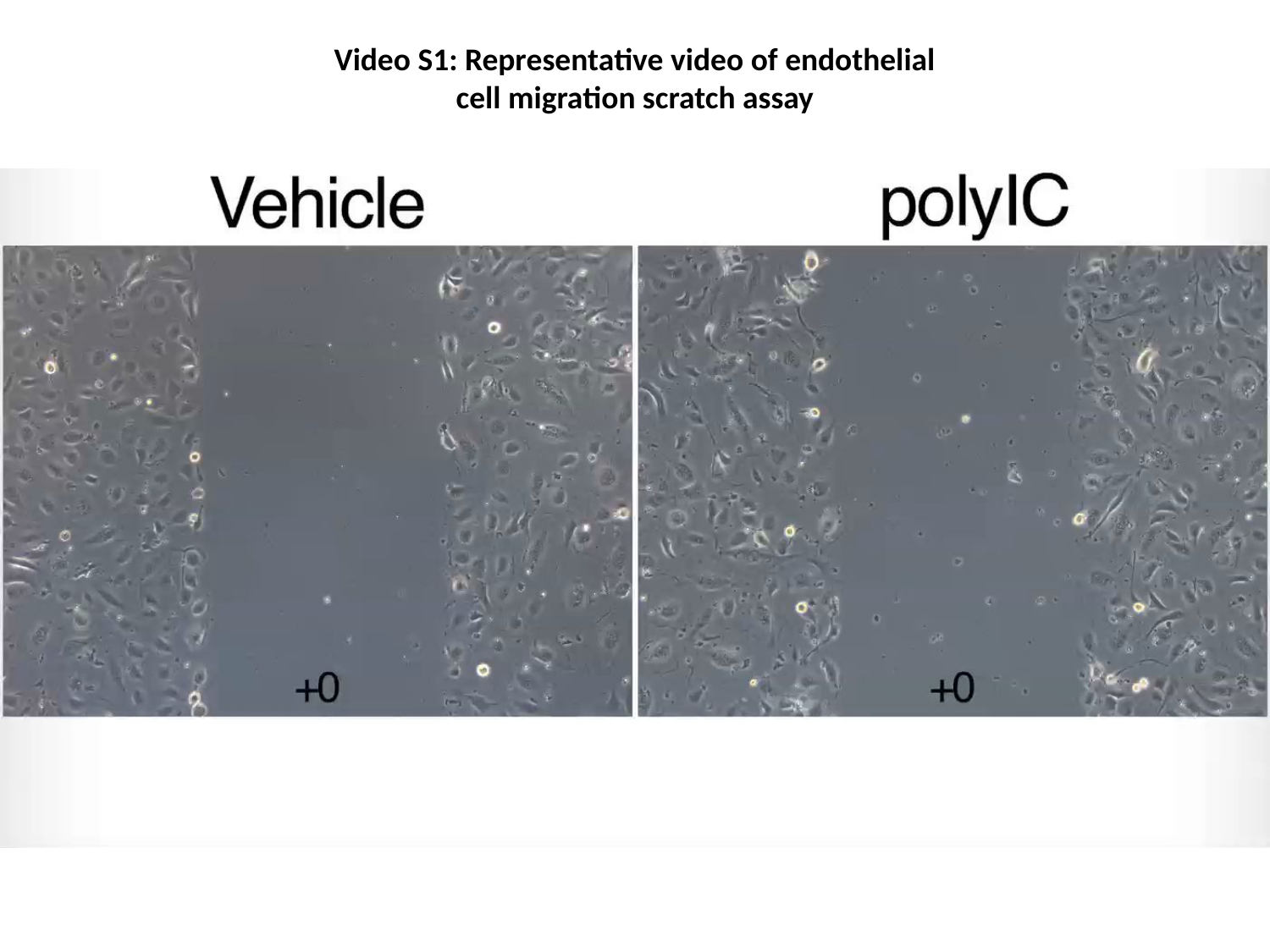

Video S1: Representative video of endothelial cell migration scratch assay

Supplement: Supplementary file 2 — Video S1 Representative video of endothelial cell migration scratch assay. The video is a composition of sequential photographs taken every 3 min. after physically scratching a cell‐free strip. Vehicle (left) and polyIC (right) stimulated cells. [file JCMM-20-1696-s002.pptx]
